# Reprogramming the immune microenvironment in lung cancer

**DOI:** 10.3389/fimmu.2025.1684889

**Published:** 2025-09-29

**Authors:** Kai Chen, Linqi Luo, Yong Li, Ge Yang

**Affiliations:** ^1^ Department of Laboratory Medicine, The Affiliated Hospital, Southwest Medical University, Luzhou, China; ^2^ The Laboratory Department of the Second People’s Hospital of Neijiang, Neijiang, China; ^3^ Neijiang Vocational College of Health and Wellness, Neijiang, China; ^4^ Department of Medical Laboratory, Dongxing District People’s Hospital of Neijiang, Neijiang, China

**Keywords:** TAM, MDSC, immune evasion, tumor microenvironment, lung cancer, immunotherapy

## Abstract

Lung cancer remains the leading cause of cancer-related mortality worldwide, with its progression shaped not only by tumor-intrinsic factors but also by a complex and immunosuppressive tumor microenvironment (TME). Within this niche, diverse immune populations—including CD8^+^ cytotoxic T cells, CD4^+^ helper T cell subsets (Th1, Th17, Tregs), B cells, natural killer (NK) cells, tumor-associated macrophages (TAMs), and myeloid-derived suppressor cells (MDSCs)—collectively regulate immune surveillance and tumor escape. While effector lymphocytes mediate antitumor responses, their function is often attenuated by TAM- and MDSC-driven immunosuppression via cytokines (IL-10, TGF-β), metabolic disruption, and immune checkpoint expression. High densities of M2-polarized TAMs and MDSCs correlate with poor prognosis and resistance to therapy. Immune checkpoint inhibitors targeting PD-1/PD-L1 and CTLA-4 have improved outcomes in lung cancer, yet therapeutic efficacy remains limited by the immunosuppressive TME. This review outlines the functional roles of key immune cell subsets in lung cancer and highlights emerging strategies to reprogram the TME and enhance immunotherapeutic responsiveness.

## 1 Introduction

Lung cancer remains the foremost contributor to global cancer-related mortality (1). Beyond intrinsic tumor cell behavior, its pathogenesis and progression are orchestrated by the surrounding tumor microenvironment (TME), a dynamic and multifaceted ecosystem that governs neoplastic proliferation, immune evasion, and metastatic competence (2, 3). The pulmonary TME comprises not only transformed epithelial cells but also a diverse milieu of stromal and immune components, including fibroblasts, vascular and lymphatic elements, extracellular matrix molecules, and a repertoire of cytokines and chemokines (4). Among these, immune constituents such as tumor-associated macrophages (TAMs) (5), dendritic cell populations (6), myeloid-derived suppressor cells (MDSCs) (7), and tumor-infiltrating lymphocytes (TILs) (8) play central roles. Their functional plasticity enables either antitumor cytotoxicity or tumor-promoting activities, including suppression of effector immunity, support for tumor growth, and facilitation of metastatic spread (9).

The therapeutic modulation of immune cells within the TME has redefined treatment paradigms for advanced pulmonary malignancies. Immune checkpoint inhibitors, particularly those targeting PD-1/PD-L1 and CTLA-4, have yielded durable responses and improved survival outcomes in subsets of patients (10). Meanwhile, emerging strategies, such as cytokine-based therapies and tumor vaccines, are under active investigation, offering new prospects for immune reprogramming (11). This review synthesizes current insights into biological functions of principal immune cell populations within lung cancer, highlighting their potential for translation into immunotherapeutic applications.

## 2 Tumor-infiltrating lymphocytes in the lung cancer

Tumor-infiltrating lymphocytes (TILs) represent a heterogeneous population of adaptive immune cells predominantly localized within the stromal regions of lung tumors, mobilized via antigen-specific responses to tumor-derived neoantigens. These cells are central to antitumor immunity, contributing to both immune surveillance and cytolytic elimination of malignant epithelial cells. Key subsets include cytotoxic CD8^+^ T cells, CD4^+^ helper T cells, regulatory T cells (Tregs), and tumor-infiltrating B cells (TIL-Bs), each exerting distinct immunoregulatory functions within the dynamic tumor microenvironment (12, 13) (**Table 1**).

**Table 1:** Tumor-Infiltrating Lymphocytes (TILs) subsets in the lung cancer microenvironment.

TIL subset	Key functions	Cytokine profile	Impacts	Clinical relevance
CD4^+^ T cells (Th1)	Activate cytotoxic T lymphocytes (CTLs) and macrophages. Promote anti-tumor immunity.	IFN-γ, TNF-α	Suppress tumor progression by promoting cell-mediated immunity.	Lower Th1/Th2 ratio correlates with poor prognosis.
CD4^+^ T cells (Th2)	Enhance humoral immunity, suppress cytotoxic T cell activity.	IL-4, IL-10	Promote tumor progression by inducing immune tolerance.	Increased Th2 cells associated with poor prognosis.
CD8^+^ T cells (CTLs)	Directly kill tumor cells via cytotoxicity and cytokine secretion.	IFN-γ, TNF-α	Strong anti-tumor effect, impaired by immune checkpoints.	Higher CTL numbers correlate with better treatment response.
Tregs	Suppress immune responses and promote tumor progression.	IL-10, TGF-β, IL-35	Promote immune evasion and tumor growth.	High Treg infiltration correlates with poor prognosis.
Th17 cells	Promote inflammation and anti-tumor immunity, but can enhance angiogenesis.	IL-17A, IL-17F, IL-21, IL-22	Inhibit tumor growth through immune responses, but can aid tumor adaptation under certain conditions.	High IL-17 expression in tissues correlates with better survival in some cases.

### 2.1 Roles of CD4^+^ T cells in lung cancer progression

#### 2.1.1 Th1 cells in the lung cancer

Th1 cells, a subset of CD4^+^ T lymphocytes, are activated upon MHC class II–restricted antigen recognition by professional antigen-presenting cells, initiating transcriptional programs that culminate in pro-inflammatory cytokine production (14). Within the TME, Th1 cells diverge into classical and Th1-like subsets with distinct effector profiles, and their balance is essential for sustaining antitumor immunity (15). The canonical Th1–Th2 paradigm delineates opposing immune trajectories. Th1 cells orchestrating cytotoxic responses and Th2 cells mediating humoral immunity, yet this dichotomy is frequently disrupted in lung cancer due to tumor-induced immune reprogramming (12). Crosstalk between neoplastic epithelial cells and helper T cell subsets often skews this balance toward a Th2-dominant state, thereby fostering a permissive TME (12). Upon activation, Th1 cells secrete IFN-γ and TNF-α, which augment antigen presentation, recruit cytotoxic cells, suppress angiogenesis, and trigger tumor cell apoptosis. Conversely, Th2 cytokines such as IL-4 and IL-10 dampen cell-mediated responses and promote tolerance (16). In lung cancer, Th2 cells are enriched within the TME compared to adjacent lung tissue, correlating with tumor grade and invasiveness. In contrast, decreased Th1 infiltration and a reduced Th1/Th2 ratio associate with immune dysfunction and disease progression (17). Notably, the immunosuppressive cytokine IL-10 not only favors Th2 polarization but also activates STAT3 signaling in dendritic cells, leading to downregulation of MHC class II expression and impaired costimulatory signaling. This diminishes antigen presentation capacity and directly attenuates Th1 priming and expansion. As a result, the IL-10/STAT3 axis plays a pivotal role in disabling effective Th1-driven antitumor responses and contributes to an immunoevasive tumor phenotype. Additionally, Tumor-derived factors such as TGF-β, IL-10, and VEGF further reinforce this immunosuppressive axis, subverting effector T cell function and establishing an immune-privileged niche conducive to lung cancer recurrence and metastasis (18). These dynamic shifts in CD4^+^ T cell plasticity represent critical determinants of lung cancer immunobiology and suggest promising avenues for therapeutic intervention.

#### 2.1.2 Th17 cells and regulatory T cells in lung carcinogenesis

Within the immune ecosystem of lung cancer, T helper 17 (Th17) cells and regulatory T cells (Tregs) exert immunologically antagonistic functions. Th17 cells orchestrate pro-inflammatory responses through the secretion of IL-17A, IL-17F, IL-21, and IL-22, which bolster local immunity and potentiate antitumor surveillance (19). Mechanistic studies indicate that IL-17 enhances dendritic cell (DC) maturation and activates CD8^+^ cytotoxic T lymphocytes (CTLs), facilitating tumor antigen clearance (20–22). Clinically, high intratumoral IL-17 expression correlates with improved progression-free and overall survival in lung cancer patients, highlighting its prognostic value (23). However, IL-17 also exhibits tumor-promoting properties in certain contexts by inducing VEGF expression and promoting neovascularization, thus fostering tumor growth and metastatic spread (24, 25). This pro-angiogenic function of IL-17 partially explains its tumor-promoting effects observed in specific microenvironmental settings. Thus, the dichotomous nature of Th17 activity reflects its context-specific function in the dynamic TME.

**Figure 1:**
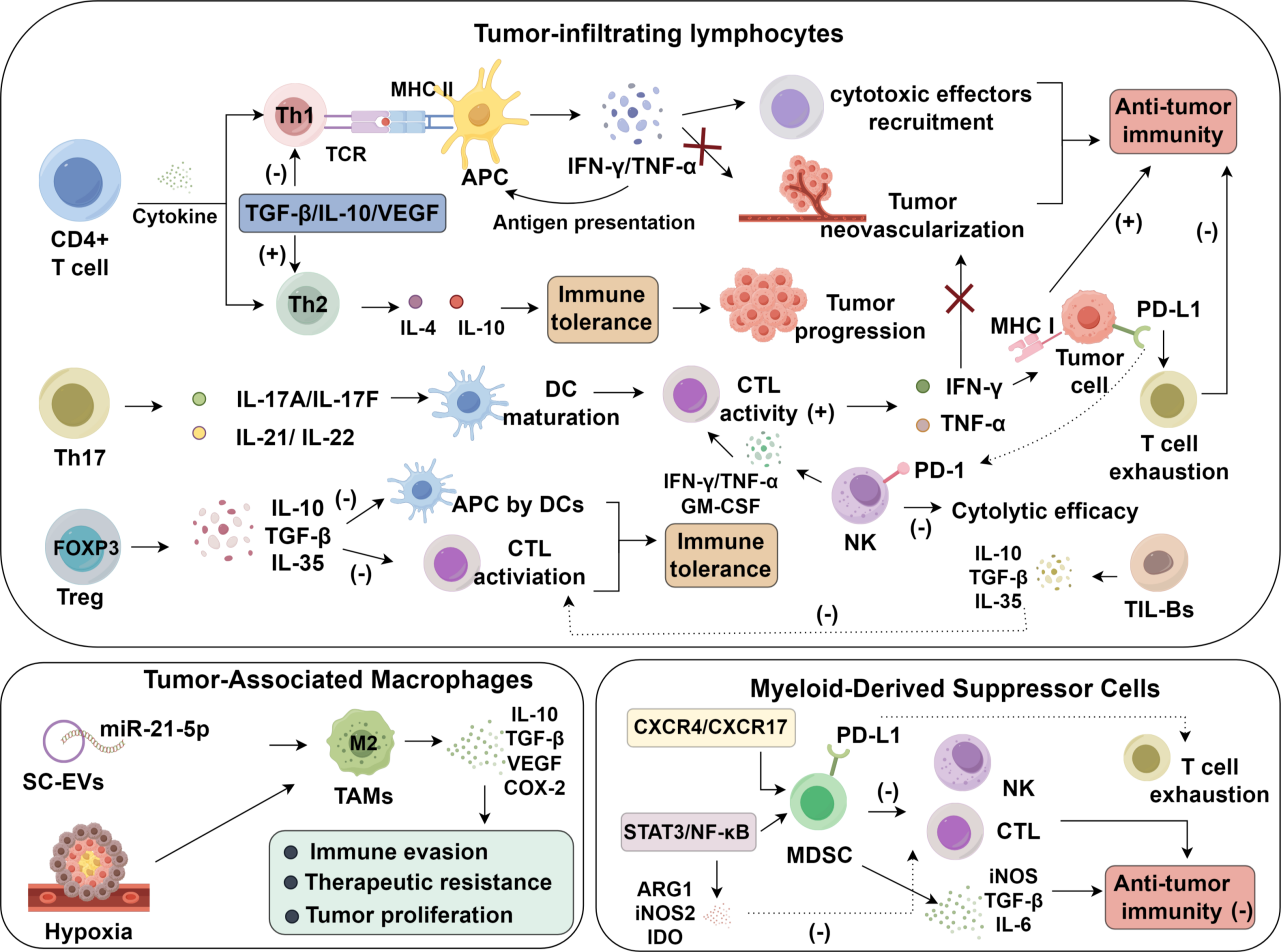
Roles of immune cell dysfunction in lung cancer.

In contrast, FOXP3^+^ Tregs critically suppress antitumor immunity by dampening CTL function and impairing antigen presentation through the secretion of IL-10, TGF-β, and IL-35 (26–29). These suppressive factors attenuate antigen presentation by DCs, downregulate costimulatory signaling, and blunt effector T cell priming, leading to an immunologically inert environment (30). In addition to cytokine secretion, FOXP3^+^ Tregs suppress antitumor responses through CTLA-4–dependent mechanisms, whereby CTLA-4 engagement downregulates CD80/CD86 expression on dendritic cells, thereby diminishing their capacity to deliver essential costimulatory signals to naïve T cells. Clinically, increased intratumoral Treg infiltration is inversely associated with patient survival outcomes (31). Thus, the opposing roles of Th17 and Treg cells highlight a delicate immunological balance in lung carcinogenesis. Their functional dichotomy provides a compelling rationale for therapeutic interventions aimed at restoring immune equilibrium, either by amplifying Th17-mediated responses or selectively targeting Treg-mediated suppression (32) (**Figure 1**).

### 2.2 Roles of CD8^+^ cytotoxic T lymphocytes in lung cancer

Cytotoxic CD8^+^ T lymphocytes (CTLs) are pivotal mediators of adaptive immunity, executing antigen-specific killing of tumor cells via recognition of tumor-associated antigens (TAAs) presented by MHC class I molecules. Upon activation, CTLs release perforin and granzymes to induce apoptosis in target cells (33, 34), and secrete pro-inflammatory cytokines such as IFN-γ and TNF-α, which collectively reinforce antitumor immunity and suppress neovascularization (35–37). IFN-γ further upregulates MHC-I expression on tumor cells, enhancing immune visibility. Notably, clinical studies have established a positive correlation between the density of tumor-infiltrating CTLs and favorable outcomes in patients with non-small cell lung cancer (38, 39), underscoring their prognostic and therapeutic significance. Nonetheless, the cytotoxic potential of CTLs is frequently compromised by immune evasion strategies within the TME. Inhibitory checkpoint molecules such as PD-1 and CTLA-4, upregulated upon T cell activation, are engaged by ligands like PD-L1 expressed on tumor and stromal cells, inducing T cell exhaustion and diminishing effector function (40, 41). Additionally, suppressive elements in the TME, including regulatory T cells, TGF-β, and IL-10, attenuate CTL proliferation and survival. To circumvent these immunosuppressive pathways, current immunotherapies, particularly immune checkpoint inhibitors targeting PD-1/PD-L1 and CTLA-4, aim to restore CTL activity in lung cancer (42, 43). Moreover, combination strategies involving checkpoint blockade, cancer vaccines, cytokine supplementation, or costimulatory agonists are under investigation to potentiate CTL responses in lung cancer treatment (44).

### 2.3 Natural killer cells: mechanisms of action and dysregulation in lung cancer

Natural killer (NK) cells are pivotal effectors of the innate immune system, acting as frontline defenders against oncogenic transformation through antigen-independent cytotoxicity. Residing predominantly in peripheral blood and secondary lymphoid tissues such as the spleen, NK cells execute immunosurveillance by detecting stress-induced ligands, such as MICA/B and ULBPs, via activating receptors including NKG2D, NKp30, and NKp46 (45). Upon engagement, NK cells unleash cytotoxic granules loaded with perforin and granzymes, perforating tumor cell membranes and initiating caspase-dependent apoptosis (46, 47). Beyond granule-mediated killing, NK cells execute apoptosis via death receptor pathways. By expressing ligands such as FasL and TRAIL, NK cells activate extrinsic apoptosis through Fas and TRAIL receptors on target cells, thereby contributing to immune clearance of transformed epithelium (48). In addition to direct cytolysis, NK cells secrete IFN-γ, TNF-α, and granulocyte-macrophage colony-stimulating factor (GM-CSF), which synergistically enhance dendritic cell maturation and potentiating CD8^+^ T cell–mediated cytotoxicity (49, 50). Moreover, NK cells facilitate antibody-dependent cellular cytotoxicity (ADCC) through CD16 (FcγRIII)-mediated recognition of antibody-opsonized tumor cells, further amplifying antitumor immunity (51).

Despite their inherent cytotoxic capabilities, NK cells are functionally compromised within the lung cancer microenvironment due to a constellation of immunosuppressive cues. Tumor-secreted factors, including TGF-β, IL-10, and prostaglandin E2 (PGE2), interfere with NK cell activation, suppress the synthesis and release of key effector molecules such as IFN-γ and perforin, and downregulate expression of activating receptors like NKG2D, collectively diminishing antitumor functionality (52, 53). Additionally, lung tumors elevate the expression of immunoinhibitory ligands, such as PD-L1 and HLA-G, which engage suppressive receptors including PD-1, NKG2A, and various KIR family members on NK cells, thereby attenuating their cytolytic efficacy (54–56). Hypoxic conditions within the tumor core further impair NK cell function by upregulating hypoxia-inducible factor 1α (HIF-1α), which alters NK cell metabolism and reduces granule-mediated killing efficiency (57–59). Moreover, tumors exploit ectonucleotidases CD39 and CD73 to generate extracellular adenosine, which accumulates in the hypoxic TME and binds to A2A adenosine receptors on NK cells. This adenosinergic signaling pathway profoundly suppresses NK cell cytotoxicity by downregulating perforin and granzyme production, impairing cytokine secretion, and inhibiting target cell lysis. These suppressive signals transform the TME into a hostile landscape for NK cell activity that facilitates immune escape and tumor progression. To restore NK cell function, therapeutic strategies targeting immunosuppressive axes, such as blockade of TGF-β and IL-10 or stimulation with IL-15/IL-21, have shown promise in preclinical models (60). Immune checkpoint inhibitors targeting NK-specific pathways, including anti-NKG2A antibody Monalizumab, have demonstrated robust potential to reinvigorate NK responses (61). Meanwhile, adoptive cellular therapy utilizing chimeric antigen receptor-engineered NK cells (CAR-NK) has emerged as a transformative modality. CAR-NK cells engineered to express tumor-targeting constructs—such as HER2-directed chimeric receptors—demonstrate enhanced tumor recognition and cytotoxicity against lung cancer cells, representing a novel frontier in innate immune-based immunotherapy (62).

### 2.4 Functional dichotomy of tumor-infiltrating B cells in lung cancer

Tumor-infiltrating B cells (TIL-Bs) constitute a heterogeneous immune population within the lung cancer microenvironment, exhibiting both immunostimulatory and immunosuppressive activities. On the one hand, TIL-Bs can enhance antitumor immunity by producing tumor-specific immunoglobulins that mediate antibody-dependent cellular cytotoxicity (ADCC), presenting tumor-derived antigens to CD4^+^ T helper cells, and facilitating the activation of CD8^+^ cytotoxic T lymphocytes (63, 64). These functions potentiate adaptive immune responses and are associated with favorable clinical outcomes, including prolonged disease-free survival and reduced recurrence in lung cancer patients (12, 65, 66). However, not all B cell subsets are protective. A specialized subpopulation termed regulatory B cells (Bregs) has been identified as a driver of immunosuppression and tumor progression. Bregs exert their immunoregulatory effects primarily through the secretion of anti-inflammatory cytokines, including IL-10, TGF-β, and IL-35, thereby impairing T cell–mediated cytotoxicity and promoting immune evasion (67). Notably, IL-35 has been shown to not only suppress CD8^+^ T cell effector functions but also actively promote the expansion and stability of Tregs, establishing a feedforward loop that reinforces the immunosuppressive tumor microenvironment. In addition to their immunoregulatory secretome, Bregs may directly enhance tumorigenesis through contact-dependent mechanisms that support tumor cell survival and proliferation (68). This IL-35–Breg–Treg axis is a critical driver of immune evasion in lung cancer. The immunological balance between effector TIL-Bs and immunosuppressive Bregs is therefore critical in shaping the trajectory of tumor–immune dynamics in lung cancer. Immunomodulatory therapies that selectively augment TIL-B activity or neutralize Breg-derived cytokines represent promising avenues for future development in lung cancer immunotherapy (69).

## 3 Tumor-associated macrophages in lung cancer invasion and immunosuppression

Tumor-associated macrophages (TAMs) represent a predominant immune subset within the lung tumor microenvironment and act as pivotal orchestrators linking chronic inflammation to oncogenesis. These cells display remarkable phenotypic plasticity, transitioning between classically activated M1-like states—associated with tumor suppression—and alternatively activated M2-like phenotypes that facilitate tumor progression. In lung cancer, TAMs are predominantly polarized toward the M2 phenotype, which promotes immune evasion, invasion, and therapeutic resistance through secretion of key immunoregulatory factors including IL-10, TGF-β, VEGF, and COX–2 (70). The polarization toward an M2 phenotype is chiefly driven by IL-4 and IL-13 which engage the IL-4Rα chain and activate STAT6. Upon activation, STAT6 translocates to the nucleus to induce expression of M2-associated genes including Arg1, Mrc1 (CD206), and Ym1, thereby establishing a transcriptional program that promotes tissue remodeling, immune suppression, and tumor tolerance. This IL-4/IL-13/STAT6 axis is a central pathway in the immunosuppressive reprogramming of macrophages within the lung tumor microenvironment.

Under hypoxic stress, macrophages increase the expression of HIF-1α, IL-10, and VEGF, thereby creating an immune-refractory, pro-angiogenic microenvironment that enhances tumor adaptability and resistance (71, 72). Notably, mesenchymal stem cell–derived extracellular vesicles (MSC-EVs) have been implicated in promoting M2 polarization through delivery of miR-21-5p. This miRNA-mediated signaling cascade enhances angiogenesis and accelerates tumor proliferation, contributing to the aggressive phenotype of lung carcinoma (73). Clinically, M2 TAM abundance correlates with unfavorable prognosis in lung cancer, whereas reduced M2 infiltration is linked to improved survival and enhanced therapeutic responsiveness (74). These findings support the development of macrophage-targeted interventions, including agents that reprogram M2 TAMs into M1-like phenotypes or disrupt key immunosuppressive axes such as IL-10 or VEGF signaling.

## 4 Myeloid-derived suppressor cells and immune tolerance in lung cancer

MDSCs, a phenotypically and functionally diverse group of immature myeloid progenitors, expand dramatically in response to chronic inflammation and tumor-derived cues within the lung cancer microenvironment. These cells are key enforcers of immunosuppression, dismantling anti-tumor immune surveillance and promoting malignant progression. MDSCs compromise cytotoxic T cell function through several coordinated mechanisms: they inhibit T cell proliferation, induce apoptosis via arginase-1 (ARG1), inducible nitric oxide synthase (iNOS), and ROS, and suppress the activation of effector T lymphocytes through multiple checkpoints (75, 76). In addition to dampening T cell responses, MDSCs foster the expansion and stabilization of regulatory T cells (Tregs) by producing immunosuppressive cytokines, particularly IL-10 and TGF-β. They also deprive T cells of L-arginine by overexpressing ARG1, which leads to downregulation of CD3ζ chain and cyclin D3, both essential for T cell receptor signaling and cell cycle progression. Furthermore, the release of nitric oxide (NO) interferes with JAK3/STAT5 signaling, while peroxynitrite (ONOO^-^), a reactive nitrogen species, modifies amino acids on TCRs, blunting T cell-mediated recognition of tumor antigens (77).

Beyond T cells, MDSCs suppress the cytolytic capacity of natural killer (NK) cells and impair B lymphocyte functions, including antibody production and clonal expansion. These suppressive effects are largely mediated by iNOS activity and prostaglandin E2 (PGE2), both of which modulate cellular activation thresholds and cytokine output (78). Clinically, elevated MDSC frequencies in tumor tissue or peripheral blood have been associated with diminished responses to chemotherapy and shorter overall survival in patients with lung malignancies (79, 80). Their recruitment is governed by chemotactic axes such as CCL2 and CXCR4, while sustained STAT3 and NF-κB activation reinforces their immunosuppressive phenotype (81, 82), upregulating enzymes including ARG1, iNOS, and indoleamine 2,3-dioxygenase (IDO), alongside anti-inflammatory cytokines that collectively suppress CTL function (83). Given their pivotal role in immune evasion, MDSCs represent compelling targets for immunomodulatory therapy. Strategies aimed at simultaneously inhibiting MDSC function and restoring T cell activity have shown promising preclinical results. Notably, dual inhibition of the C5aR1 and the PD-1/PD-L1 axis has been shown to enhance anti-tumor immune responses, leading to reduced tumor burden and decreased metastatic dissemination (84, 85). Likewise, co-administration of MEK inhibitors with immune checkpoint inhibitors augments infiltration of CD8^+^ and CD4^+^ T cells, enhances antigen-specific responses, and prolongs survival in murine models of lung cancer (85).

An abundance of MDSCs in the lung cancer setting inversely correlates with therapeutic efficacy, particularly in the context of chemotherapy and immunotherapy (86). Their suppressive activity directly interferes with the ability of T and NK cells to mount effective anti-tumor responses, thereby compromising clinical outcomes (87). This immunosuppressive program is mediated through upregulation of iNOS, TGF-β, and IL-6, alongside Treg induction and expansion (88–91). The trafficking of MDSCs into tumor tissues is driven by gradients of chemokines and corresponding receptors—including CXCR4 and CXCR17—which direct their accumulation in the tumor bed (81, 82, 92). Persistent activation of STAT3 and NF-κB transcription factors reinforces the MDSC phenotype, promoting the expression of immunosuppressive mediators such as ARG1, iNOS2, IDO, and cytokines that suppress T cell effector function (93). Notably, PD-L1 expression on MDSCs plays a central role in mediating T cell exhaustion and immune escape in lung cancer, highlighting their intersection with immune checkpoint pathways (94, 95). Dual targeting of PD-1 and either the C5a/C5aR1 or MEK pathway has shown superior efficacy in preclinical models, leading to enhanced T cell recruitment and sustained suppression of tumor growth. These findings provide a strong rationale for integrated treatment regimens that concurrently target immune checkpoints and myeloid-derived suppressors to optimize outcomes in lung cancer therapy.

## 5 Conclusion

Lung cancer progression is shaped not only by intrinsic tumor biology but also by a profoundly immunosuppressive microenvironment, wherein tumor-associated macrophages, myeloid-derived suppressor cells, regulatory T cells, and dysfunctional NK and B cells coordinate to dampen antitumor immunity. Although immune checkpoint blockade has improved survival in subsets of patients, its efficacy remains limited by persistent immunosuppressive signals, metabolic dysfunction, and cellular exhaustion within the tumor niche. Emerging strategies—such as dual inhibition of PD-1/PD-L1 and myeloid-derived pathways (C5aR1, STAT3), reprogramming of TAM and Treg phenotypes, and adoptive cell therapies—offer a promising path toward immune reinvigoration and durable clinical response.

However, several limitations remain. Most preclinical models fail to fully recapitulate the complexity and heterogeneity of the human TME, which limits translational predictability. Moreover, the dynamic plasticity of immune cell subsets and inter-patient variability in immune landscapes pose challenges for biomarker development and therapy stratification. Future research should focus on spatially resolved, single-cell, and multi-omics profiling of the TME to decipher patient-specific immunological architecture. Integrating these insights with longitudinal clinical data will be critical for developing precision immunotherapies that can circumvent resistance, restore immune surveillance, and ultimately transform lung cancer management.
